# Associations between diagnoses linked with adverse COVID-19-related outcomes and sickness absence due to COVID-19 or COVID-19 like diagnoses: a prospective Swedish cohort study of 292 274 blue-collar workers in the retail and wholesale industry

**DOI:** 10.1093/eurpub/ckaf177

**Published:** 2025-11-07

**Authors:** Lukasz Cybulski, Emma Pettersson, Kristina Alexanderson, Kristin Farrants

**Affiliations:** Division of Insurance Medicine, Department of Clinical Neuroscience, Karolinska Institutet, Stockholm, Sweden; Division of Insurance Medicine, Department of Clinical Neuroscience, Karolinska Institutet, Stockholm, Sweden; Division of Insurance Medicine, Department of Clinical Neuroscience, Karolinska Institutet, Stockholm, Sweden; Division of Insurance Medicine, Department of Clinical Neuroscience, Karolinska Institutet, Stockholm, Sweden

## Abstract

Several diagnoses have been associated with increased risk for adverse health outcomes following COVID-19 infection. Whether these diagnoses also predispose individuals to long-term sickness absence (SA) due to COVID-19 is unclear. The aim was to examine associations between risk diagnoses and long-term SA due to COVID-19/COVID-19-like diagnoses among blue-collar workers in the retail and wholesale industry. We conducted a two-year prospective cohort study of all blue-collar workers aged 18–67 in 2019, employed in retail and wholesale industry in Sweden (*N* = 292 274), using linked microdata from several nationwide registers. We estimated odds ratios (OR) through logistic regression models to determine the association between diagnoses linked with adverse health outcomes following COVID-19 infection and SA spells due to COVID-19/COVID-19-like diagnoses in 2020–2021. We included COVID-19-like diagnoses because of diagnostic uncertainty early in the pandemic. Of all the workers, 34 594 (11.8%) had least one COVID-19 risk diagnosis. Only 7812 (2.7%) workers had SA due to COVID-19/COVID-19-like diagnoses in the two-year follow-up (2020–2021). Most risk diagnoses were associated with elevated likelihoods of SA due to COVID-19, particularly immunodeficiency (OR 2.58; 95% CI 1.68–3.27) and respiratory disease (2.00; 1.89–2.12). The associations for these diagnoses and diabetes and hypertension persisted after we stratified by prior SA, but no other risk diagnosis was significantly associated with SA due to COVID-19/COVID-19-like diagnoses among those with prior SA in 2019. Stratifying on all-cause SA thus removed the association between most COVID-19 risk diagnoses and SA due to COVID-19/COVID-19-like diagnoses.

## Introduction

Sickness absence (SA) increased markedly during the COVID-19 pandemic relative to previous years [1–3]. There is now a growing body of literature characterizing the patterns and risks for SA due to COVID-19 during this period, with the healthcare sector receiving particular attention given the elevated risks for exposure that its workers faced during this period [4–6]. Much less is known about blue-collar workers in retail and wholesale, who may have been subject to similarly challenging circumstances during the pandemic. For example, many workers in this group were considered indispensable and could not work remotely, and there are now studies demonstrating elevated risks for COVID-19 infection in several blue-collar occupations, including retail workers [7–10]. Moreover, research predating the pandemic has shown that blue-collar workers generally tend to have higher rates of SA relative to white-collar workers [11, 12]. Multiple studies also suggest that the incidence of common disorders linked to disability and premature death, such as cardiovascular disease, cancer, and diabetes, is higher in many blue-collar occupations relative to white-collar occupations, including workers in service professions, transport, and the retail and wholesale industries [13–16]. As the pandemic progressed it became evident that many of these diagnoses predispose individuals infected with COVID-19 to worse outcomes, including a more protracted and disabling course of illness and an elevated risk for death [17]. The National Board of Health and Welfare, Sweden’s central authority for social and health services, developed a list of diagnoses associated with particularly severe trajectories, including cardiovascular disease, cancer, autoimmune disorders, and obesity [18]. To date, there is no published research on the extent to which these diagnoses were associated with COVID-19-related SA among blue-collar workers in retail and wholesale. Since many of these risk diagnoses may be prevalent among blue-collar workers and because of the potentially higher risks of exposure to COVID-19 inherent to many blue-collar occupations in retail and wholesale, this population may have been especially affected during the pandemic. Most people with a COVID-19 infection had a relatively short disease duration, with few having SA for more than 14 days [19]. However, it is unclear if this was the case among individuals with risk diagnoses for severe COVID-19 infections. Therefore, the aim was to examine the risk of future SA spells >14 days due to COVID-19/COVID-19-like diagnoses among blue-collar workers in retail and wholesale who had a prior risk diagnosis for poor health outcomes following COVID-19 infection.

## Methods

We conducted a prospective cohort study utilizing interlinked microdata from several nationwide Swedish administrative registers [20].

### Data and study population

Information on sociodemographic and occupational factors were obtained from Statistics Sweden’s Longitudinal Integrated Database for Health Insurance and Labour Market Studies (LISA) [21]. Data on SA, including the SA diagnosis, were sourced from the Swedish Social Insurance Agency’s Micro Data for Analyses of Social Insurance (MiDAS) [22]. We used two registers provided by the Swedish National Board of Health and Welfare to obtain information about our study population’s medical histories: (i) The National Patient Database, which contains information about diagnoses and treatments received within specialized (secondary) in- or outpatient healthcare, and (ii) the National Prescribed Drug register, which includes data on all prescribed drugs dispensed at pharmacies. The latter was used because some conditions that we were interested in often are treated with medication in primary care (e.g. diabetes, asthma, hypertension, etc.).

We included individuals aged 18–67 in 2019, who also were registered as residents of Sweden in 2020, and who were employed in private-sector companies in retail and wholesale in blue-collar occupations, as defined by the Swedish Standard for Occupational Classification (SSYK) [23]. We considered individuals to be employed if they had income from work, parental benefits, and/or SA that amounted to at least 8370 SEK per year (∼790 EUR; 2019 exchange rate) (i.e. 75% of the necessary income level to qualify for SA benefits from the Social Insurance Agency, in order not to miss anyone) [24]. We excluded individuals who were self-employed, employed in the public sector, or who had full-time disability pension (DP) throughout the entirety of 2020–2021.

### Public sickness absence benefit insurance in Sweden

In Sweden, individuals whose income from work, parental leave, or unemployment benefits exceed a minimum threshold are eligible for SA benefits if their work capacity is reduced due to morbidity. Following an initial waiting day, employers are legally obliged to provide sick pay during the subsequent 13 days. If the SA spell exceeds 14 days, the responsibility for compensation is transferred onto the Social Insurance Agency. Unemployed individuals get SA benefits from the Social Insurance Agency from the second day. Thus, we only included SA spells covered by the Social Insurance Agency that lasted a minimum of 14 days. SA benefits cover 80% of lost income, up to a certain level, and can be granted on a part-time or full-time basis (i.e. 25%, 50%, 75%, or 100% of ordinary working hours). Thus, individuals can be on part-time SA at the same time and for this reason we calculated net days. For example, two gross days of 50% absence were combined to one net day.

### Outcomes

We used codes from the 10th edition of the International Classification of Disease (ICD-10) to ascertain outcomes and exposures. The outcome measure in this study was having a new recorded SA spell during 2020–2021, either due to COVID-19 (U07 or U10), post-COVID-19 viral fatigue (U09 or G933), or due to related diagnoses. This latter category included certain diseases of the respiratory system (J00, J02, J04, J06, J11, J12, J16, J18, J20, J21, J22, J44, J45, J46, J80, J96, J98); certain infectious and parasitic diseases (A08, A09, B09, B34, B97, B99), and the following symptoms, signs, and abnormal clinical and laboratory findings, not elsewhere classified (R00, R05, R06, R07, R20, R21, R23, R43, R50, R51, R53, R65). We followed the practice of the Swedish Social Insurance Agency and used the latter, more inclusive definition because testing for COVID-19 was less prevalent in the early stages of the pandemic and many individuals who had SA due to COVID-19 were, therefore, ascribed diagnoses with similar symptoms [19]. We excluded individuals who had been granted DP due to COVID-19/COVID-19-like diagnoses because there were few cases (<10) and because they all were on long-term DP with COVID-19-like diagnoses in 2019.

### Exposures

Exposure information was sourced from the National Patient Register, the MIDAS register, and the Prescribed Drug Register. The primary exposure was having a recorded diagnosis associated with either prior treatment for, or a prior SA spell, due to any of the following 16 diagnoses in 2016–2019: cancer (C00–C97), heart disease (I50, I27, I34, I35, I36, I37, I38, I39, Q23), hypertension (I10–I15), stroke (I63), diabetes (E10–E11), renal disease (N10–N19), liver disorders (B18, K70, K71, K74), respiratory disease (E84, J40–J47, J84), neurological disorders affecting respiratory function (G10–G14, G35), obesity (E66), other disorders of the adrenal gland (E27), organ transplant (Z94), immunodeficiency (D80–D89), Human Immunodeficiency Virus (HIV) (B20–B24), Down syndrome (Q90), severe mental disorders (F20, F31). We also utilized Anatomical Therapeutic Chemical (ATC) codes to ascertain diagnoses of conditions that are routinely treated in primary care through medication, including diabetes, hypertension, and respiratory disease (See Supplementary Table S1 for an exhaustive list of ATC-codes). We decided to focus on these 16 diagnostic categories as they previously have been identified as risk factors for severe COVID-19 outcomes by the Swedish National Board of Health and Welfare [18]. In addition to examining the association between these diagnoses and SA due to COVID-19/COVID-19-like diagnoses separately, we also constructed a binary composite exposure measure of having any of these diagnoses. For certain diagnoses, including liver disorders, HIV, and Down syndrome, the number of observations was very low. To protect the confidentiality of affected individuals we did not derive individual estimates for these conditions. Instead, they were included in the overall measure of risk indicators for severe COVID-19.

### Covariates

We also examined an array of sociodemographic characteristics as covariates, including sex; age (18–24, 25–34, 35–44, 45–54, 55–64, or 65–67 years); country of birth [Sweden, other Nordic country, other EU27, or rest of world, including missing values (*n* = 25; 0%)]; educational level [elementary (<10 years including missing values (*n* = 2235 (0.8%)), high school (10–12 years), or university/college (>12 years)]; family situation (married/cohabiting with children below the age of 18 at home, married/cohabiting without children at home, single with children at home, or single without children at home); type of place of residence according to Eurostat’s Degree of Urbanisation (DEGURBA) [25] (city, town or suburb, or rural area); type of occupation based on the SSYK [23] categorized into the following 15 groups: (sales assistant for daily goods, sales assistant for specialist trade, construction workers, craft workers, cashiers, warehouse and terminal staff, machine and process operators, mechanics, technicians, repair, installation etc., motor vehicle mechanics and repair personnel, transport occupations, security staff, porters, cleaners, etc., other logistics, other service staff, other sales staff, and other occupations). We also examined if individuals had SA due to any cause in 2019 that lasted for 14 days or more.

### Statistical analyses

We estimated odds ratios (OR) and 95% confidence intervals (CI) through multivariable logistic regression models to examine the strength of association between sociodemographic (i.e. sex, age, educational level, family situation, country of birth, type of place of residence, occupation), and risk diagnoses (i.e. prior diagnosis of cancer, cardiovascular disease, hypertension, diabetes, renal disease, respiratory disease, neurological disorders, obesity, organ transplantation, immunodeficiency, and severe mental disorders), and SA due to COVID-19/COVID-19-like diagnoses. We fitted crude, partially adjusted (sex, age, educational level, family situation, country of birth, type of place of residency), and fully adjusted (i.e. for all other sociodemographic variables and having a recorded COVID-19 risk diagnosis in 2016–2019) models. Because prior SA itself is a strong predictor for future SA [26, 27], we stratified analyses on prior SA due to any cause in 2019. Analyses were performed in R version 4.3.1.

## Results

In total, our cohort included 292 274 individuals, of which 139 551 (47.7%) were women (Table 1). SA due to COVID-19/COVID-19-like diagnoses was relatively rare in our cohort, with only 7873 (2.7%) individuals having a spell during 2020 and 2021. In 2020, 1491 (27%) of SA spells were due COVID-19 with the remaining 73% being accounted for by COVID-19-like diagnoses. The proportion of SA spells due to COVID-19 increased the following year, to 36.5% (Supplementary Table S2). In terms of clinical characteristics, 34 594 (11.8%) individuals had at least one risk diagnosis for more severe COVID-19 sequelae, with half of them being some type of cancer (6.0%). As can be seen in Table 2, all risk diagnoses that we examined were consistently associated with increased odds for SA due to COVID-19/COVID-19-like diagnoses, except for heart disease, which only was associated with elevated odds for SA due to COVID-19 or COVID-19-like diagnoses in crude models. In our models adjusted for sociodemographic factors, immunodeficiency (OR 2.58, 95% CI 1.55–3.27), having undergone an organ transplant (2.47, 1.61–3.79), and respiratory disease (2.0, 1.89–2.12) emerged as the three diagnostic categories that had the strongest association with SA due to COVID-19/COVID-19-like diagnoses. However, the former two diagnoses were very rare, occurring in just 31 (0.01%) and 23 (0.007%) individuals, respectively. Those diagnosed with a severe mental disorder (1.87, 1.46–2.40), obesity (1.85, 1.59–2.15), or diabetes (1.67, 1.30–1.62) were also more likely to experience SA due to COVID-19/COVID-19-like diagnoses, also after we adjusted for sociodemographic variables. When we stratified these associations on prior SA in 2019 due to any cause, we observed an attenuation of associations among individuals with prior SA. In the stratified models adjusted for sociodemographic factors, only hypertension, diabetes, respiratory disease, and immunodeficiency were associated with SA due to COVID-19/COVID-19-like diagnoses during follow-up, and in the fully adjusted model, autoimmune disease was no longer associated with SA (Table 3). By contrast, the associations in the group with no prior SA resembled the pattern observed in the main analysis of all individuals.

**Table 1.** Cohort characteristics

Characteristic in 2019	Overall (*N*, %)
	*N* = 292 754
Sex	
Female	139 551 (47.7%)
Male	153 203 (52.3%)
Age	
18–24	77 838 (26.6%)
25–34	90 296 (30.8%)
35–44	48 657 (16.6%)
45–54	43 534 (14.9%)
55–64	29 588 (10.1%)
65–67	2841 (1.0%)
Educational level	
University/college (>12 years)	53 835 (18.4%)
High school (10–12 years)	198 482 (67.8%)
Elementary school (<10 years)	40 437 (13.8%)
Family situation	
Single, no children <18 years at home	176 742 (60.4%)
Single, children <18 years at home	10 038 (3.4%)
Partner, no children at home	34 396 (11.7%)
Partner, children <18 years at home	71 578 (24.4%)
Region of birth	
Sweden	247 871 (84.7%)
Other nordic countries	3063 (1.0%)
Rest of Europe (EU27)	7237 (2.5%)
Rest of the world + missing	34 583 (11.8%)
Type of place of residence	
Rural areas	53 564 (18.3%)
Towns/suburbs	125 113 (42.7%)
Cities	114 077 (39.0%)
Number of SA days (2019)	
0	268 269 (91.6%)
1–30	8721 (3.0%)
31–90	10 208 (3.5%)
91–180	3968 (1.4%)
181–365	1588 (0.5%)
Occupation	
Sales assistant for daily goods	76 680 (26.2%)
Sales assistant for specialist trade	92 074 (31.5%)
Construction workers	6108 (2.1%)
Craft workers	3107 (1.1%)
Cashiers, etc	5873 (2.0%)
Warehouse and terminal staff	34 046 (11.6%)
Machine and process operators	3482 (1.2%)
Mechanics, technicians, repair, installation, etc	11 302 (3.9%)
Motor vehicle mechanics and repair personnel	21 908 (7.5%)
Transport occupations	4491 (1.5%)
Security staff, porters, cleaners, etc	6565 (2.2%)
Other logistics	5953 (2.0%)
Other service staff	12 616 (4.3%)
Other sales staff	5992 (2.0%)
Other occupation	2557 (0.9%)
Risk diagnoses for severe Covid-19	
Respiratory disease	31 886 (10.9%)
Hypertension	22 572 (7.7%)
Malign cancer	17 496 (6.0%)
Diabetes	7110 (2.4%)
Obesity	3468 (1.2%)
Renal disease	2163 (0.7%)
Severe mental disorder	1351 (0.5%)
Heart disease	1149 (0.4%)
Neurological disorders	465 (0.2%)
Immunodeficiency	416 (0.1%)
Stroke	278 (0.1%)
Transplant	300 (0.1%)
At least one risk diagnosis	71 888 (24.6%)
SA due to Covid-19 and/or related diagnoses in 2020 or 2021	
No	284 881 (97.3%)
Yes	7873 (2.7%)

**Table 2.** Odds ratios showing the association between Covid-19 risk diagnosis and sickness absence due to Covid-19 or related diagnoses in 2020–2021

	SA events (*N* = 7873)		Crude OR (95% CI)	Partially adjusted^a^ OR (95% CI)	Fully adjusted^b^ OR (95% CI)
Covid-19 risk diagnoses	With risk factor *n* (%)	Without risk factor *n* (%)			
Respiratory disease	1528 (4.8%)	6345 (2.4%)	2.02 (1.91–2.14)	2.00 (1.89–2.12)	1.93 (1.82–2.04)
Hypertension	1239 (5.5%)	6634 (2.5%)	2.31 (2.17–2.46)	1.58 (1.48–1.69)	1.41 (1.31–1.51)
Malign cancer	795 (4.5%)	7 078 (2.6%)	1.80 (1.67–1.94)	1.47 (1.36–1.58)	1.40 (1.30–1.51)
Diabetes	375 (5.3%)	7 498 (2.6%)	2.07 (1.86–2.30)	1.57 (1.41–1.75)	1.29 (1.15–1.44)
Obesity	186 (5.4%)	7 687 (2.7%)	2.08 (1.79–2.41)	1.85 (1.59–2.15)	1.55 (1.33–1.81)
Renal disease	85 (3.9%)	7 788 (2.7%)	1.49 (1.19–1.85)	1.43 (1.15–1.78)	1.14 (0.91–1.43)
Severe mental disorder	66 (4.9%)	7 807 (2.7%)	1.87 (1.46–2.39)	1.87 (1.46–2.40)	1.81 (1.41–2.32)
Heart disease	44 (3.8%)	7 829 (2.7%)	1.44 (1.07–1.95)	1.18 (0.87–1.59)	0.85 (0.63–1.16)
Immunodeficiency	31 (7.5%)	7 842 (2.7%)	2.92 (2.02–4.21)	2.58 (1.78–3.74)	1.86 (1.28–2.70)
Neurological disorders	24 (5.2%)	7 849 (2.7%)	1.97 (1.31–2.98)	1.63 (1.08–2.47)	1.55 (1.03–2.36)
Transplant	23 (7.7%)	7 850 (2.7%)	3.01 (1.97–4.61)	2.47 (1.61–3.79)	1.69 (1.08–2.63)
Stroke	18 (6.5%)	7 855 (2.7%)	2.51 (1.56–4.05)	1.66 (1.02–2.68)	1.38 (0.85–2.24)
At least one risk diagnosis	3 207 (4.5%)	4 666 (2.1%)	2.16 (2.07–2.26)		1.82 (1.74–1.91)

a Each risk factor was modelled separately; each model was adjusted for sex, age, educational level, family situation, region of birth, and type of living area.

b Adjusted for all variables listed in model a and for having a diagnosis of any of the other COVID-19 risk diagnoses during 2016–2019.

**Table 3.** Odds ratios showing the association between Covid-19 risk diagnosis and SA, stratified by having had prior all-cause sickness absence (SA) during 2019

			Crude	Partially adjusted^a^	Fully adjusted^b^
Covid-19 risk diagnoses	With risk factor *n* (%)	Without risk factor *n* (%)	OR (95% CI)	OR (95% CI)	OR (95% CI)
With SA in 2019	SA events (*N* = 1713)				
Respiratory disease	429 (10.8%)	1 284 (6.3%)	1.82 (1.62–2.04)	1.77 (1.58–1.99)	1.73 (1.54–1.95)
Hypertension	381 (10.1%)	1 332 (6.4%)	1.63 (1.45–1.84)	1.38 (1.21–1.58)	1.29 (1.13–1.49)
Malign cancer	235 (8.5%)	1 478 (6.8%)	1.28 (1.11–1.48)	1.13 (0.97–1.30)	1.11 (0.95–1.28)
Diabetes	117 (10.6%)	1 596 (6.8%)	1.61 (1.32–1.97)	1.41 (1.15–1.73)	1.26 (1.02–1.55)
Obesity	69 (8.3%)	1 644 (7.0%)	1.21 (0.94–1.56)	1.21 (0.94–1.56)	1.08 (0.83–1.40)
Severe mental disorder	33 (8.5%)	1 680 (7.0%)	1.24 (0.87–1.78)	1.31 (0.91–1.89)	1.34 (0.93–1.93)
Renal disease	27 (6.9%)	1 686 (7.0%)	0.99 (0.66–1.46)	0.99 (0.66–1.47)	0.85 (0.56–1.29)
Heart disease	22 (9.3%)	1 691 (7.0%)	1.36 (0.88–2.12)	1.27 (0.81–1.98)	1.04 (0.66–1.65)
Neurological disorders	12 (11.9%)	1 701 (7.0%)	1.80 (0.98–3.29)	1.68 (0.91–3.09)	1.58 (0.85–2.92)
Immunodeficiency	12 (14.3%)	1 701 (7.0%)	2.22 (1.20–4.11)	2.13 (1.15–3.97)	1.72 (0.92–3.21)
Transplant	11 (11.6%)	1 702 (7.0%)	1.75 (0.93–3.28)	1.71 (0.90–3.23)	1.48 (0.76–2.88)
Stroke	10 (8.6%)	1 703 (7.0%)	1.26 (0.66–2.41)	1.06 (0.55–2.03)	0.97 (0.50–1.87)
At least one risk diagnosis	901 (9.0%)	812 (5.6%)	1.65 (1.50–1.83)		1.49 (1.35–1.65)
Without SA in 2019	SA events (*N* = 6160)				
Respiratory disease	1099 (3.9%)	5061 (2.1%)	1.98 (1.68–2.34)	2.26 (1.91–2.67)	2.11 (1.78–2.50)
Hypertension	858 (4.6%)	5302 (2.1%)	2.28 (1.96–2.65)	1.53 (1.31–1.78)	1.25 (1.06–1.48)
Malign cancer	560 (3.8%)	5600 (2.2%)	1.75 (1.60–1.91)	1.46 (1.33–1.59)	1.42 (1.30–1.56)
Diabetes	258 (4.3%)	5902 (2.3%)	1.77 (1.48–2.11)	1.54 (1.29–1.84)	1.35 (1.12–1.62)
Obesity	117 (4.4%)	6043 (2.3%)	2.00 (1.65–2.41)	1.84 (1.52–2.22)	1.66 (1.37–2.01)
Renal disease	58 (3.3%)	6102 (2.3%)	1.44 (1.11–1.88)	1.43 (1.09–1.86)	1.22 (0.93–1.60)
Severe mental disorder	33 (3.4%)	6127 (2.3%)	1.51 (1.07–2.14)	1.57 (1.11–2.23)	1.54 (1.08–2.19)
Heart disease	22 (2.4%)	6138 (2.3%)	1.05 (0.69–1.61)	0.86 (0.56–1.32)	0.69 (0.45–1.06)
Immunodeficiency	19 (5.7%)	6141 (2.3%)	2.59 (1.63–4.11)	2.30 (1.44–3.67)	1.94 (1.21–3.10)
Neurological disorders	12 (3.3%)	6148 (2.3%)	1.45 (0.82–2.58)	1.20 (0.68–2.15)	1.19 (0.67–2.12)
Transplant	12 (5.9%)	6148 (2.3%)	2.65 (1.48–4.75)	2.18 (1.21–3.92)	1.56 (0.85–2.84)
Stroke	<10 (4.9%)	6152 (2.3%)	2.21 (1.09–4.50)	1.43 (0.70–2.92)	1.28 (0.62–2.62)
At least one risk diagnosis	2306 (3.7%)	3854 (1.9%)	1.82 (1.70–1.95)		1.58 (1.48–1.69)

a Each risk factor was modelled separately; each model was adjusted for sex, age, educational level, family situation, region of birth, and type of living area.

b Adjusted for all variables listed in model a and for having a diagnosis of any of the other COVID-19 risk diagnoses during 2016–2019.

Table 4 shows crude and adjusted ORs for having SA due to COVID-19/COVID-19-like diagnoses by sociodemographic characteristics. After adjusting for socioeconomic factors, men had lower odds of SA due to COVID-19/COVID-19-like diagnoses than women (0.76, 0.72–0.80). The likelihood of SA due to COVID-19/COVID-19-like diagnoses increased incrementally with age, up until the oldest age group (65–67), who were less likely than the reference category (25–34) to have an equivalent SA spell. Most occupational categories were relative to Sales assistant for specialist trade goods neither less nor more likely to have SA due to COVID-19/COVID-19-like diagnoses, although there were some exceptions to this.

**Table 4.** Distribution of sociodemographic characteristics and odds ratios showing their association with SA due to Covid-19 or related diagnoses

Characteristics in 2019	SA due to Covid-19 or related diagnoses		Model	
	No	Yes	Crude	Adjusted^a^
	N = 284881 (97.3%)	N = 7873 (2.69%)	OR (95% CI)	OR (95% CI)
Sex				
Female	135 532 (97.1%)	4 019 (2.9%)	Ref	Ref
Male	149 349 (97.5%)	3 854 (2.5%)	0.87 (0.83–0.91)	0.76 (0.72–0.80)
Age				
18–24	77 001 (98.9%)	837 (1.1%)	0.50 (0.46–0.54)	0.50 (0.46–0.55)
25–34	88 381 (97.9%)	1,915 (2.1%)	Ref	Ref
35–44	46 930 (96.5%)	1,727 (3.5%)	1.70 (1.59–1.81)	1.55 (1.45–1.67)
45–54	41 632 (95.6%)	1 902 (4.4%)	2.11 (1.98–2.25)	1.88 (1.76–2.02)
55–64	28 146 (95.1%)	1 442 (4.9%)	2.36 (2.21–2.54)	2.02 (1.86–2.19)
65–67	2 791 (98.2%)	50 (1.8%)	0.83 (0.62–1.10)	0.67 (0.50–0.89)
Years of education				
University/college (>12 years)	52 690 (97.9%)	1 145 (2.1%)	Ref	Ref
High school (10–12 years)	193 148 (97.3%)	5 334 (2.7%)	1.27 (1.19–1.36)	1.30 (1.22–1.39)
Elementary school (<10 years)	39 043 (96.6%)	1 394 (3.4%)	1.64 (1.52–1.78)	1.43 (1.32–1.56)
Family situation				
Single, no children <18 years at home	173 035 (97.9%)	3 707 (2.1%)	Ref	Ref
Single, children <18 years at home	9 641 (96.0%)	397 (4.0%)	1.92 (1.73–2.14)	1.15 (1.03–1.29)
Partner, no children at home	32 944 (95.8%)	1 452 (4.2%)	2.06 (1.93–2.19)	1.00 (0.93–1.08)
Partner, children <18 years at home	69 261 (96.8%)	2 317 (3.2%)	1.56 (1.48–1.65)	1.07 (1.01–1.13)
Country of birth				
Sweden	241 759 (97.5%)	6 112 (2.5%)	Ref	Ref
Norden (except Sweden)	2 952 (96.4%)	111 (3.6%)	1.49 (1.23–1.80)	1.05 (0.87–1.28)
EU27 (except Denmark, Finland and Sweden)	7 004 (96.8%)	233 (3.2%)	1.32 (1.15–1.50)	1.22 (1.06–1.40)
Rest of the world	33 166 (95.9%)	1 417 (4.1%)	1.69 (1.59–1.79)	1.56 (1.46–1.66)
Type of place of residence				
Small town or rural area	52 210 (97.5%)	1 354 (2.5%)	Ref	Ref
Medium-size town	121 857 (97.4%)	3 256 (2.6%)	1.03 (0.97–1.10)	1.08 (1.01–1.16)
Large city	110 814 (97.1%)	3 263 (2.9%)	1.14 (1.06–1.21)	1.26 (1.18–1.35)
Occupation				
Sales assistant for specialist trade	89 700 (97.4%)	2 374 (2.6%)	Ref	Ref
Sales assistant for daily goods	74 676 (97.4%)	2 004 (2.6%)	1.01 (0.95–1.08)	1.01 (0.95–1.08)
Warehouse and terminal staff	32 726 (96.1%)	1 320 (3.9%)	1.52 (1.42–1.63)	1.40 (1.30–1.51)
Motor vehicle mechanics and repair personnel	21 368 (97.5%)	540 (2.5%)	0.95 (0.87–1.05)	0.95 (0.86–1.06)
Other service staff	12 331 (97.7%)	285 (2.3%)	0.87 (0.77–0.99)	0.98 (0.87–1.11)
Mechanics, technicians, repair, installation, etc	11 030 (97.6%)	272 (2.4%)	0.93 (0.82–1.06)	0.90 (0.79–1.03)
Security staff, porters, cleaners, etc	6 419 (97.8%)	146 (2.2%)	0.86 (0.73–1.02)	0.73 (0.61–0.87)
Construction workers	5 942 (97.3%)	166 (2.7%)	1.06 (0.90–1.24)	0.98 (0.83–1.15)
Other sales staff	5 857 (97.7%)	135 (2.3%)	0.87 (0.73–1.04)	0.94 (0.78–1.12)
Other logistics	5 798 (97.4%)	155 (2.6%)	1.01 (0.86–1.19)	0.94 (0.79–1.11)
Cashiers	5 716 (97.3%)	157 (2.7%)	1.04 (0.88–1.22)	1.04 (0.88–1.23)
Transport occupations	4 367 (97.2%)	124 (2.8%)	1.07 (0.89–1.29)	0.94 (0.78–1.13)
Machine and process operators	3 402 (97.7%)	80 (2.3%)	0.89 (0.71–1.11)	0.78 (0.62–0.99)
Craft workers	3 039 (97.8%)	68 (2.2%)	0.85 (0.66–1.08)	0.74 (0.58–0.94)
Other occupations	2 510 (98.2%)	47 (1.8%)	0.71 (0.53–0.95)	0.64 (0.48–0.86)

a Adjusted for sex, age, educational level, family situation, country of birth, and type of place of residence.

## Discussion

In this nationwide prospective cohort study of all blue-collar workers employed in retail and wholesale businesses in Sweden, we explored if risk diagnoses for severe outcomes following COVID-19 infection were associated with future SA due to COVID-19/COVID-19-like diagnoses. Our findings show that only a small minority (2.7%) of workers had SA due to COVID-19/COVID-19-like diagnoses during 2020–20201. Individuals with a recorded risk diagnosis were more likely to have SA due to COVID-19/COVID-19-like diagnoses, particularly those with a diagnosis of respiratory disease and immunodeficiency. However, in analyses stratified by prior all-cause SA in 2019, most risk diagnoses ceased to be associated with an increased likelihood of SA due to COVID-19/COVID-19-like diagnoses. In terms of sociodemographic characteristics and their association with SA, our results followed a familiar pattern, with men, individuals born in Sweden, and those with higher education being less likely to have SA due to COVID-19/COVID-19-like diagnoses.

Existing research linking diagnoses to SA due to COVID-19 is relatively scarce and to the best of our knowledge this has not been examined among blue-collar workers in retail and wholesale. Direct comparisons with investigations are also complicated by the heterogeneity of studies in terms of study design and the diagnoses that they examined. Nevertheless, comparisons are possible with two previous studies [28, 29]. For instance, a Swedish cohort study of 661 780 individuals diagnosed with COVID-19 found that 5.7% had SA due to COVID-19 > 14 days, and that cardiovascular disease, respiratory disease, cancer, or diabetes were more prevalent among them [29]. Workers with these diagnoses also had higher odds for SA due to COVID-19 in our study. Similarly, work by Abzhandadze and colleagues found diagnoses of respiratory disease to be associated with higher odds for prolonged SA spells (>84 days) among those on SA due to COVID-19.

Studies that examine the association between diagnoses and COVID-19-related health outcomes are by contrast much more abundant and our findings are broadly consistent with this literature. There is now a wealth of research implicating respiratory diseases, autoimmune disorders, cancer, obesity, diabetes, organ transplant, and cardiovascular disease with several adverse COVID-19-related health outcomes, including a more protracted course of disease [30], hospitalization [31], and death [17]. The higher risk for more protracted or severe COVID-19 infections associated with these diagnoses may explain why affected workers were more likely to experience SA due to COVID-19/COVID-19-like diagnoses. With respect to specific diagnoses, respiratory disease and autoimmune disorders stood out as the two diagnoses associated with the highest odds for SA. This is not surprising because COVID-19 primarily affects the respiratory system [32], and some studies have reported comparatively higher risks for mortality due to COVID-19 associated with respiratory disease, such as chronic obstructive pulmonary disease (COPD) [17]. Similarly, immunodeficiency has also been associated with a more severe and longer course of disease [33]. However, it is worth noting that some of the diagnoses strongly associated with SA due to COVID-19-like diagnoses, such as immunodeficiency or organ transplant, were very uncommon in our study sample.

Whilst some of these diagnoses appear to increase the likelihood of SA due to COVID-19/COVID-19-like diagnoses, it is worth pointing out that most individuals in our cohort (88.2%) did not have a history of such diagnoses. In addition, many of the associations that we observed between risk diagnoses and COVID-19-related SA were no longer significant after we restricted our analysis to those who had a history of SA due to any cause in 2019. More specifically, among individuals without prior SA in 2019, the results mirrored those in the entire study cohort, with most diagnoses being associated with SA due to COVID-19. However, this was not the case among those with SA in 2019. Thus, it appears that prior SA due to any cause often is a similarly strong indicator for future SA due to COVID-19/COVID-19-like diagnoses as risk diagnoses for adverse COVID-19-related health outcomes. The notable exceptions to this were respiratory disease, immunodeficiency, diabetes, and hypertension, which continued to be associated with COVID-19-related SA among individuals with prior SA, which may reflect that these diagnoses have particularly severe interactions with COVID-19. Indeed, several Swedish studies looking at the characteristics of patients admitted to hospital following COVID-19 infection have found diabetes, hypertension, and respiratory disease to be the most prevalent pre-existing diagnoses in this group [34–36]. However, other diagnoses linked with adverse COVID-19-related outcomes, such as cancer, were not associated with elevated odds for SA due to COVID-19/COVID-19-like diagnoses when compared with those with prior SA. Because we adjusted our analyses for sex, age, occupation, and other sociodemographic factors, it is unlikely that differences in the distribution of these characteristics explain the higher odds for SA due to COVID-19/COVID-19-like diagnoses that we observed for respiratory disease, immunodeficiency, diabetes, and hypertension. These results could, however, reflect differences in the distribution of some other factor that we could not account for.

In terms of sociodemographic characteristics, our results followed a pattern that is familiar [24, 37–39]. That is, women exhibited higher odds for SA than men, as did individuals with lower educational level and those born outside of Sweden. We also saw that those in the oldest age group (65–67) were less likely to have SA, which could be due to higher proportion of individuals in this age group choosing to retire instead of being sickness absent. Most individuals in this group will also have had access to vaccination in early 2021, which is likely to have lowered the likelihood of severe COVID-19 infection. However, studies that have examined health-related outcomes associated with COVID-19 consistently observe a heightened risk for adverse outcomes among men. Thus, whilst there clearly is a lot of overlap between the literature on health-related COVID-19 outcomes and SA due to COVID-19/COVID-19-like diagnoses with respect to how these two outcomes relate to pre-existing diagnoses, there are also notable differences, and in some respects our findings align more closely to those observed in studies that have focused on SA [28].

### Strengths and weaknesses

Our study had several strengths. For instance, because our study utilized linked high-quality microdata with nationwide coverage, we could delineate a cohort consisting of nearly all blue-collar workers in retail and wholesale in Sweden. The exception to this is the relatively few workers in the retail sector who are employed publicly. Our results are therefore representative of nearly the entire Swedish blue-collar population working in retail and wholesale. Moreover, because we used linked data from multiple registers, we had access to a rich array of sociodemographic and employment-related information with no loss to follow-up. These administrative data are also collected routinely and are therefore not subject to biases inherent to self-reported data. Whilst the datasets we utilized provide nationwide coverage for healthcare use and SA, diagnosis of COVID-19 was unreliable in the early phases of the pandemic and many affected individuals who presented to healthcare services were assigned other diagnoses. To address this issue, we implemented a more inclusive outcome definition. Whilst this approach may have allowed us to capture SA due to COVID-19 that were not formally diagnosed, it is also likely that we included individuals on SA due to COVID19-like-diagnoses who did not have SA due to COVID-19, thereby introducing noise into our estimates. Moreover, in some individuals the associations between exposure and outcome may therefore reflect a continuation a non-COVID-19 disease.

## Conclusions

Risk diagnoses for severe health outcomes following COVID-19 infection also appears to be linked with elevated odds for SA spells >14 days due to COVID-19/COVID-19-like diagnoses. However, our study showed that SA due to any cause often is a similarly strong indicator for subsequent SA due to COVID-19/COVID-19-like diagnoses as having a risk diagnosis for more severe COVID-19-related health outcomes. The exceptions to this were respiratory disease, diabetes, hypertension, and immunodeficiency, which were associated with higher likelihoods of SA due to COVID-19, even when compared with those who had prior SA due to other diagnoses. Whilst the risk diagnoses we examined in this study appear to be significant risk factors, our study showed that they are relatively uncommon, with the vast majority of blue-collar workers in the trade and retail industries being unaffected. Moreover, SA due to COVID-19/COVID-19-like diagnoses was relatively rare, with only 2.69% of the study population having a COVID-19-related spell across 2020 and 2021, meaning that the overall impact of the COVID-19 pandemic was relatively small in terms of SA spells longer than 14 days, which is consistent with analyses of the entire working population [19]. Future studies could explore these aspects in other occupational sectors, which may vary considerably in terms of working culture and conditions.

## Supplementary Material

ckaf177_Supplementary_Data

## Data Availability

The data used in this study cannot be made publicly available due to privacy regulations. According to the General Data Protection Regulation, the Swedish law SFS 2018:218, the Swedish Data Protection Act, the Swedish Ethical Review Act, and the Public Access to Information and Secrecy Act, these types of sensitive data can only be made available for specific purposes that meet the criteria for access to this type of sensitive and confidential data as determined by a legal review. Contact imas-cns@ki.se for information regarding the data. Key points Only a small minority of blue-collar workers in retail and wholesale in Sweden had sickness absence (SA) due to COVID-19 or COVID-19-like diagnoses in 2020–2021. One in 10 workers had *a prior* risk diagnosis for adverse COVID-19-related health outcomes. SA due to any cause often is a similarly strong indicator for subsequent SA due to COVID-19 as having a risk diagnosis for more severe COVID-19-related health outcomes.

## References

[1] van der Plaat DA , Edge R, Coggon D et al Impact of COVID-19 pandemic on sickness absence for mental ill health in National Health Service staff. BMJ Open 2021;11:e054533. 10.1136/bmjopen-2021-054533 PMC8572362 34732501

[2] Smith DRM , Jijón S, Oodally A et al Sick leave due to COVID-19 during the first pandemic wave in France, 2020. Occup Environ Med 2023;80:268–72. 36914254 10.1136/oemed-2022-108451 PMC10176331

[3] Calvo-Bonacho E , Catalina-Romero C, Fernández-Labandera C et al COVID-19 and sick leave: an analysis of the ibermutua cohort of over 1,651,305 Spanish workers in the first trimester of 2020. Front Public Health 2020;8:580546. 33194983 10.3389/fpubh.2020.580546 PMC7604328

[4] Reme BA , Grosland M, Gjefsen H et al Impact of the COVID-19 pandemic on sick leave among healthcare workers: a register-based observational study. Occup Environ Med 2023;80:319–25. 37068949 10.1136/oemed-2022-108555 PMC10314089

[5] Kisiel MA , Nordqvist T, Westman G et al Patterns and predictors of sick leave among Swedish non-hospitalized healthcare and residential care workers with Covid-19 during the early phase of the pandemic. PLoS One 2021;16:e0260652. 34882720 10.1371/journal.pone.0260652 PMC8659339

[6] van der Plaat DA , Madan I, Coggon D et al Risks of COVID-19 by occupation in NHS workers in England. Occup Environ Med 2022;79:176–83. 34462304 10.1136/oemed-2021-107628

[7] Office of National Statistics. Which occupations have the highest potential exposure to the coronavirus (COVID-19)?. https://www.ons.gov.uk/employmentandlabourmarket/peopleinwork/employmentandemployeetypes/articles/whichoccupationshavethehighestpotentialexposuretothecoronaviruscovid19/2020-05-11. 2020.

[8] Reuter M , Rigó M, Formazin M et al Occupation and SARS-CoV-2 infection risk among 108 960 workers during the first pandemic wave in Germany. Scand J Work Environ Health 2022;48:446–56. 35670286 10.5271/sjweh.4037 PMC9888438

[9] Magnusson K , Nygard K, Methi F et al Occupational risk of COVID-19 in the first versus second epidemic wave in Norway, 2020. Euro Surveill 2021;26:2001875. 34622761 10.2807/1560-7917.ES.2021.26.40.2001875 PMC8511752

[10] de Gier B , de Oliveira Bressane Lima P, van Gaalen RD et al Occupation- and age-associated risk of SARS-CoV-2 test positivity, The Netherlands, June to October 2020. Euro Surveill 2020;25:2001884. 33334396 10.2807/1560-7917.ES.2020.25.50.2001884 PMC7812419

[11] Väänänen A , Kalimo R, Toppinen-Tanner S et al Role clarity, fairness, and organizational climate as predictors of sickness absence: a prospective study in the private sector. Scand J Public Health 2004;32:426–34. 15762027 10.1080/14034940410028136

[12] Piha K , Laaksonen M, Martikainen P et al Interrelationships between education, occupational class, income and sickness absence. Eur J Public Health 2010;20:276–80. 19843600 10.1093/eurpub/ckp162

[13] Pukkala E , Martinsen JI, Lynge E et al Occupation and cancer—follow-up of 15 million people in five Nordic countries. Acta Oncol 2009;48:646–790. 19925375 10.1080/02841860902913546

[14] Carlsson S , Andersson T, Talback M et al Mortality rates and cardiovascular disease burden in type 2 diabetes by occupation, results from all Swedish employees in 2002–2015. Cardiovasc Diabetol 2021;20:129. 34174883 10.1186/s12933-021-01320-8 PMC8235252

[15] Krause N , Arah OA, Kauhanen J. Physical activity and 22-year all-cause and coronary heart disease mortality. Am J Ind Med 2017;60:976–90. 28940659 10.1002/ajim.22756

[16] Marmot M. Status Syndrome: How Your Social Standing Directly Affects Your Health and Life Expectancy. London: Bloomsbury Publishing, 2004.

[17] Dessie ZG , Zewotir T. Mortality-related risk factors of COVID-19: a systematic review and meta-analysis of 42 studies and 423,117 patients. BMC Infect Dis 2021;21:855. 34418980 10.1186/s12879-021-06536-3 PMC8380115

[18] Uppdatering av tidigare rapport gällande identifiering av riskgrupper som löper störst risk att drabbas av ett särskilt allvarligt sjukdomsförlopp vid insjuknande i covid-19 [Update of earlier report regarding the identification of groups with the highest risk of severe health consequences following Covid-19 infection]. The National Board of Social Affairs and Health, 2020 [in Swedish].

[19] Effekter som covid-19 har på sjukförsäkringen: Delrapport 3 [Effects of Covid-19 on social insurance—Part 3]. Swedish Social Insurance Agency, 2022 [in Swedish].

[20] Ludvigsson JF , Otterblad-Olausson P, Pettersson BU et al The Swedish personal identity number: possibilities and pitfalls in healthcare and medical research. Eur J Epidemiol 2009;24:659–67. 19504049 10.1007/s10654-009-9350-y PMC2773709

[21] Ludvigsson JF , Svedberg P, Olén O et al The longitudinal integrated database for health insurance and labour market studies (LISA) and its use in medical research. Eur J Epidemiol 2019;34:423–37. 30929112 10.1007/s10654-019-00511-8 PMC6451717

[22] Österlund N. MiDAS Sjukpenning och rehabiliteringspenning. Stockholm: Försäkringskassan, 2011.

[23] SSYK 2012 Swedish Standard Classification of Occupations. Statistics Sweden, 2012.

[24] Farrants K , Alexanderson K. Sickness absence among privately employed white-collar workers: a total population study in Sweden. Scand J Public Health 2021;49:159–67. 32650706 10.1177/1403494820934275 PMC7917566

[25] Applying the Degree of Urbanisation—A Methodological manual to define cities, towns and rural areas for international comparisons. EUROSTAT, 2021.

[26] Dekkers-Sanchez PM , Hoving JL, Sluiter JK et al Factors associated with long-term sick leave in sick-listed employees: a systematic review. Occup Environ Med 2008;65:153–7. 17881466 10.1136/oem.2007.034983

[27] Farrants K , Head J, Alexanderson K. Trends in associations between sickness absence before the age of 65 and being in paid work after the age of 65: prospective study of three total population cohorts. J Aging Soc Policy 2023;35:197–220. 35114914 10.1080/08959420.2021.2022950

[28] Abzhandadze T , Westerlind E, Persson HC. Impact of pre-pandemic sick leave diagnoses on the length of COVID-19-related sick leave: a nationwide registry-based study. BMC Public Health 2023;23:195. 36709256 10.1186/s12889-023-15115-x PMC9884157

[29] Spetz M , Natt Och Dag Y, Li H et al The sociodemographic patterning of sick leave and determinants of longer sick leave after mild and severe COVID-19: a nationwide register-based study in Sweden. Eur J Public Health 2024;34:121–8. 37889580 10.1093/eurpub/ckad191 PMC10843940

[30] Ose DJ , Gardner E, Millar M et al A cross-sectional and population-based study from primary care on post-COVID-19 conditions in non-hospitalized patients. Commun Med (Lond) 2024;4:24. 38383883 10.1038/s43856-024-00440-y PMC10881566

[31] Kompaniyets L , Pennington AF, Goodman AB et al Underlying medical conditions and severe illness among 540,667 adults hospitalized with COVID-19, March 2020–March 2021. Prev Chronic Dis. 2021;18:E66. 34197283 10.5888/pcd18.210123 PMC8269743

[32] Parasher A. COVID-19: current understanding of its pathophysiology, clinical presentation and treatment. Postgrad Med J 2021;97:312–20. 32978337 10.1136/postgradmedj-2020-138577 PMC10017004

[33] Garmendia JV , Garcia AH, De Sanctis CV et al Autoimmunity and immunodeficiency in severe SARS-CoV-2 infection and prolonged COVID-19. Curr Issues Mol Biol 2022;45:33–50. 36661489 10.3390/cimb45010003 PMC9857622

[34] Strålin K , Wahlström E, Walther S et al Mortality trends among hospitalised COVID-19 patients in Sweden: a nationwide observational cohort study. Lancet Reg Health Eur 2021;4:100054. 33997829 10.1016/j.lanepe.2021.100054 PMC7907732

[35] Lindgren M , Toska T, Alex C et al BMI, sex and outcomes in hospitalised patients in Western Sweden during the COVID-19 pandemic. Sci Rep 2022;12:4918. 35318438 10.1038/s41598-022-09027-w PMC8939489

[36] Sjostrom B , Mansson E, Viklund Kamienny J et al Characteristics and definitive outcomes of COVID-19 patients admitted to a secondary hospital intensive care unit in Sweden. Health Sci Rep 2021;4:e446. 34938894 10.1002/hsr2.446 PMC8670731

[37] Farrants K , Alexanderson K. Sickness absence and disability pension in the trade and retail industry: a prospective cohort study of 192,000 white-collar workers in Sweden. J Occup Environ Med 2022;64:912–9. 35901218 10.1097/JOM.0000000000002634 PMC9640291

[38] Allebeck P , Mastekaasa A. Swedish council on technology assessment in health care (SBU). Chapter 5. Risk factors for sick leave—general studies. Scand J Publ Health 2004;32:49–108. 10.1080/14034950410021853 15513654

[39] Beemsterboer W , Stewart R, Groothoff J et al A literature review on sick leave determinants (1984–2004). Int J Occup Med Environ Health 2009;22:169–79. 19617195 10.2478/v10001-009-0013-8

