# Supplementary material for: Associations between diagnoses linked with adverse COVID-19-related outcomes and sickness absence due to COVID-19 or COVID-19 like diagnoses: a prospective Swedish cohort study of 292 274 blue-collar workers in the retail and wholesale industry
Source: Eur J Public Health. 2025 Nov 7;35(6):1300–6. doi: 10.1093/eurpub/ckaf177 (PMC12707510; doi:10.1093/eurpub/ckaf177)
Supplement: ckaf177_Supplementary_Data [file ckaf177_supplementary_data.zip › EJPH_table_S1[AU].pdf]

Table S1. List of ATC codes use to identify treatment of diabetes

Indication	ATC	Substance
asthma	R03AC02	Salbutamol
asthma	R03AC03	Terbutalin
asthma	R03AC12	Salmeterol
asthma	R03AC13	Formoterol
asthma	R03AK06	Salmeterol/Flutikasonpropionat
asthma	R03AK07	Formoterol/Budesonid
asthma	R03AK08	Formoterol/Beklometasondipropionat (vattenfritt)
asthma	R03AK10	Vilanterol/Flutikasonfuroat
asthma	R03AK11	Formoterol/Flutikasonpropionat
asthma	R03AK14	Indakaterol
asthma	R03AL09	Formoterol/Beklometasondipropionat (vattenfritt)
asthma	R03AL12	Indakaterol/Glykopyrronium
asthma	R03BA01	Beklometason
asthma	R03BA02	Budesonid
asthma	R03BA08	Ciklesonid
asthma	R03BB01	Ipratropium
asthma	R03BB04	Tiotropium
asthma	R03CC02	Salbutamol
asthma	R03CC03	Terbutalin
copd	R03AC18	Indakaterol
copd	R03AL02	Salbutamol
copd	R03AL03	Vilanterol/Umeklidinium
copd	R03AL04	Indakaterol/Glykopyrronium
copd	R03AL05	Formoterol
copd	R03AL06	Tiotropium/Olodaterol
copd	R03AL08	Vilanterol/Flutikasonfuroat/Umeklidinium
copd	R03AL11	Budesonid/Formoterol/Glykopyrronium
diabetes_both	A10AB01	Insulin
diabetes_both	A10AB04	Insulin
diabetes_both	A10AB05	Insulin
diabetes_both	A10AB06	Insulin
diabetes_both	A10AC01	Insulin
diabetes_both	A10AD04	Insulin
diabetes_both	A10AD05	Insulin
diabetes_both	A10AE04	Insulin
diabetes_both	A10AE05	Insulin
diabetes_both	A10AE06	Insulin
diabetes_both	A10AE54	Insulin
diabetes_both	A10AE56	Insulin
diabetes_type_2	A10AE56	Liraglutid/Insulin
diabetes_type_2	A10BA02	Metformin
diabetes_type_2	A10BB12	Glimepirid

diabetes_type_2	A10BD07	Metformin/Sitagliptin
diabetes_type_2	A10BD08	Metformin/Vildagliptin
diabetes_type_2	A10BD10	Metformin/Saxagliptin
diabetes_type_2	A10BD11	Metformin/Linagliptin
diabetes_type_2	A10BD15	Dapagliflozin/Metformin
diabetes_type_2	A10BD19	Empagliflozin/Linagliptin
diabetes_type_2	A10BD20	Empagliflozin/Metformin
diabetes_type_2	A10BD21	Dapagliflozin/Saxagliptin
diabetes_type_2	A10BH05	Linagliptin
diabetes_type_2	A10BJ02	Liraglutid
diabetes_type_2	A10BJ06	Semaglutid
diabetes_type_2	A10BK01	Dapagliflozin
diabetes_type_2	A10BK03	Empagliflozin
diabetes_type_2	A10BX02	Repaglinid
diabetes_type_2	A10BX02	Repaglinid
hypertension	C02CA04	Doxazosin
hypertension	C03AA01	Bendroflumetiazid
hypertension	C03AA03	Hydroklortiazid
hypertension	C03AB01	Bendroflumetiazid/Kaliumklorid
hypertension	C03DA01	Spironolakton
hypertension	C03EA01	Hydroklortiazid/Amilorid
hypertension	C07AB02	Metoprolol
hypertension	C07AG01	Labetalol
hypertension	C07FB02	Metoprolol/Felodipin
hypertension	C08CA01	Amlodipin
hypertension	C09AA02	Enalapril
hypertension	C09AA05	Ramipril
hypertension	C09BA02	Enalapril/Hydroklortiazid
hypertension	C09BA05	Ramipril/Hydroklortiazid
hypertension	C09CA01	Losartan
hypertension	C09CA06	Kandesartan
hypertension	C09DA01	Losartan/Hydroklortiazid
hypertension	C09DA06	Kandesartan/Hydroklortiazid
hypertension	C09DB01	Amlodipin/Valsartan
