# Supplementary material for: Associations between diagnoses linked with adverse COVID-19-related outcomes and sickness absence due to COVID-19 or COVID-19 like diagnoses: a prospective Swedish cohort study of 292 274 blue-collar workers in the retail and wholesale industry
Source: Eur J Public Health. 2025 Nov 7;35(6):1300–6. doi: 10.1093/eurpub/ckaf177 (PMC12707510; doi:10.1093/eurpub/ckaf177)
Supplement: ckaf177_Supplementary_Data [file ckaf177_supplementary_data.zip › EJPH_table_S2[AU].pdf]

Table S2. Distribution of sickness absence (SA) due to COVID-19 or COVID-19-like diagnoses by ICD-10 codes and calendar year at spell level

	N (%)		
**ICD-10 codes**	**SA in 2020**	**SA in 2021**	**SA in 2020-2021**
COVID-19 (U07, U10)	1491 (27.0)	1041 (36.5)	2532 (30.2)
Post-COVID (U09, G933)	132 (2.4)	394 (13.8)	526 (6.3)
Respiratory disease (J00, J02, J04, J06, J11, J12, J16, J18, J20, J21, J22, J44, J45, J46, J80, J96, J98)	2653 (48.0)	823 (28.8)	3476 (41.4)
Infectious and parasitic diseases (A08, A09, B09, B34, B97, B99)	653 (11.8)	167 (5.9)	820 (9.8)
Symptoms, signs and abnormal clinical and laboratory findings, not elsewhere classified (R00, R05, R06, R07, R20, R21, R23, R43, R50, R51, R53, R65)	613 (11.1)	429 (15.0)	1042 (12.4)
