# Supplementary material for: Associations between diagnoses linked with adverse COVID-19-related outcomes and sickness absence due to COVID-19 or COVID-19 like diagnoses: a prospective Swedish cohort study of 292 274 blue-collar workers in the retail and wholesale industry
Source: Eur J Public Health. 2025 Nov 7;35(6):1300–6. doi: 10.1093/eurpub/ckaf177 (PMC12707510; doi:10.1093/eurpub/ckaf177)
Supplement: ckaf177_Supplementary_Data [file ckaf177_supplementary_data.zip › Supplement_Data.zip!/ejph-2024-10-om-0729-File006.docx]

| **Table S1**. List of ATC codes use to identify treatment of diabetes | | |
| --- | --- | --- |
| Indication | ATC | Substance |
|  |  |  |
| asthma | R03AC02 | Salbutamol |
| asthma | R03AC03 | Terbutalin |
| asthma | R03AC12 | Salmeterol |
| asthma | R03AC13 | Formoterol |
| asthma | R03AK06 | Salmeterol/Flutikasonpropionat |
| asthma | R03AK07 | Formoterol/Budesonid |
| asthma | R03AK08 | Formoterol/Beklometasondipropionat (vattenfritt) |
| asthma | R03AK10 | Vilanterol/Flutikasonfuroat |
| asthma | R03AK11 | Formoterol/Flutikasonpropionat |
| asthma | R03AK14 | Indakaterol |
| asthma | R03AL09 | Formoterol/Beklometasondipropionat (vattenfritt) |
| asthma | R03AL12 | Indakaterol/Glykopyrronium |
| asthma | R03BA01 | Beklometason |
| asthma | R03BA02 | Budesonid |
| asthma | R03BA08 | Ciklesonid |
| asthma | R03BB01 | Ipratropium |
| asthma | R03BB04 | Tiotropium |
| asthma | R03CC02 | Salbutamol |
| asthma | R03CC03 | Terbutalin |
| copd | R03AC18 | Indakaterol |
| copd | R03AL02 | Salbutamol |
| copd | R03AL03 | Vilanterol/Umeklidinium |
| copd | R03AL04 | Indakaterol/Glykopyrronium |
| copd | R03AL05 | Formoterol |
| copd | R03AL06 | Tiotropium/Olodaterol |
| copd | R03AL08 | Vilanterol/Flutikasonfuroat/Umeklidinium |
| copd | R03AL11 | Budesonid/Formoterol/Glykopyrronium |
| diabetes_both | A10AB01 | Insulin |
| diabetes_both | A10AB04 | Insulin |
| diabetes_both | A10AB05 | Insulin |
| diabetes_both | A10AB06 | Insulin |
| diabetes_both | A10AC01 | Insulin |
| diabetes_both | A10AD04 | Insulin |
| diabetes_both | A10AD05 | Insulin |
| diabetes_both | A10AE04 | Insulin |
| diabetes_both | A10AE05 | Insulin |
| diabetes_both | A10AE06 | Insulin |
| diabetes_both | A10AE54 | Insulin |
| diabetes_both | A10AE56 | Insulin |
| diabetes_type_2 | A10AE56 | Liraglutid/Insulin |
| diabetes_type_2 | A10BA02 | Metformin |
| diabetes_type_2 | A10BB12 | Glimepirid |
| diabetes_type_2 | A10BD07 | Metformin/Sitagliptin |
| diabetes_type_2 | A10BD08 | Metformin/Vildagliptin |
| diabetes_type_2 | A10BD10 | Metformin/Saxagliptin |
| diabetes_type_2 | A10BD11 | Metformin/Linagliptin |
| diabetes_type_2 | A10BD15 | Dapagliflozin/Metformin |
| diabetes_type_2 | A10BD19 | Empagliflozin/Linagliptin |
| diabetes_type_2 | A10BD20 | Empagliflozin/Metformin |
| diabetes_type_2 | A10BD21 | Dapagliflozin/Saxagliptin |
| diabetes_type_2 | A10BH05 | Linagliptin |
| diabetes_type_2 | A10BJ02 | Liraglutid |
| diabetes_type_2 | A10BJ06 | Semaglutid |
| diabetes_type_2 | A10BK01 | Dapagliflozin |
| diabetes_type_2 | A10BK03 | Empagliflozin |
| diabetes_type_2 | A10BX02 | Repaglinid |
| diabetes_type_2 | A10BX02 | Repaglinid |
| hypertension | C02CA04 | Doxazosin |
| hypertension | C03AA01 | Bendroflumetiazid |
| hypertension | C03AA03 | Hydroklortiazid |
| hypertension | C03AB01 | Bendroflumetiazid/Kaliumklorid |
| hypertension | C03DA01 | Spironolakton |
| hypertension | C03EA01 | Hydroklortiazid/Amilorid |
| hypertension | C07AB02 | Metoprolol |
| hypertension | C07AG01 | Labetalol |
| hypertension | C07FB02 | Metoprolol/Felodipin |
| hypertension | C08CA01 | Amlodipin |
| hypertension | C09AA02 | Enalapril |
| hypertension | C09AA05 | Ramipril |
| hypertension | C09BA02 | Enalapril/Hydroklortiazid |
| hypertension | C09BA05 | Ramipril/Hydroklortiazid |
| hypertension | C09CA01 | Losartan |
| hypertension | C09CA06 | Kandesartan |
| hypertension | C09DA01 | Losartan/Hydroklortiazid |
| hypertension | C09DA06 | Kandesartan/Hydroklortiazid |
| hypertension | C09DB01 | Amlodipin/Valsartan |
