# Supplementary material for: Associations between diagnoses linked with adverse COVID-19-related outcomes and sickness absence due to COVID-19 or COVID-19 like diagnoses: a prospective Swedish cohort study of 292 274 blue-collar workers in the retail and wholesale industry
Source: Eur J Public Health. 2025 Nov 7;35(6):1300–6. doi: 10.1093/eurpub/ckaf177 (PMC12707510; doi:10.1093/eurpub/ckaf177)
Supplement: ckaf177_Supplementary_Data [file ckaf177_supplementary_data.zip › Supplement_Data.zip!/ejph-2024-10-om-0729-File007.docx]

|  |  | N (%) |  |
| --- | --- | --- | --- |
| **ICD-10 codes** | **SA in 2020** | **SA in 2021** | **SA in 2020-2021** |
|  |  |  |  |
| COVID-19 (U07, U10) | 1491 (27.0) | 1041 (36.5) | 2532 (30.2) |
| Post-COVID (U09, G933) | 132 (2.4) | 394 (13.8) | 526 (6.3) |
| Respiratory disease (J00, J02, J04, J06, J11, J12, J16, J18, J20, J21, J22, J44, J45, J46, J80, J96, J98) | 2653 (48.0) | 823 (28.8) | 3476 (41.4) |
| Infectious and parasitic diseases (A08, A09, B09, B34, B97, B99) | 653 (11.8) | 167 (5.9) | 820 (9.8) |
| Symptoms, signs and abnormal clinical and laboratory findings, not elsewhere classified (R00, R05, R06, R07, R20, R21, R23, R43, R50, R51, R53, R65) | 613 (11.1) | 429 (15.0) | 1042 (12.4) |

**Table S2**. Distribution of sickness absence (SA) due to COVID-19 or COVID-19-like diagnoses by ICD-10 codes and calendar year at spell level
